# Core Steps to the Azaphilone Family of Fungal Natural Products

**DOI:** 10.1002/cbic.202100240

**Published:** 2021-07-09

**Authors:** Katherine Williams, Claudio Greco, Andrew M. Bailey, Christine L. Willis

**Affiliations:** ^1^ School of Biological Sciences University of Bristol Life Sciences Building, 24 Tyndall Avenue Bristol BS8 1TQ UK; ^2^ Department of Molecular Microbiology John Innes Centre Norwich NR4 7UH UK; ^3^ School of Chemistry University of Bristol Cantock's Close Bristol BS8 1TS UK

**Keywords:** azaphilone, biosynthesis, genome mining, natural products, pathway elucidation

## Abstract

Azaphilones are a family of polyketide‐based fungal natural products that exhibit interesting and useful bioactivities. This minireview explores the literature on various characterised azaphilone biosynthetic pathways, which allows for a proposed consensus scheme for the production of the core azaphilone structure, as well as identifying early diversification steps during azaphilone biosynthesis. A consensus understanding of the core enzymatic steps towards a particular family of fungal natural products can aid in genome‐mining experiments. Genome mining for novel fungal natural products is a powerful technique for both exploring chemical space and providing new insights into fungal natural product pathways.

## Graphical abstract

**Azaphilones** are a polyketide‐based family of fungal natural products with a highly oxygenated bicyclic pyrone‐quinone structure, many of which have useful and interesting bioactivities. This minireview reveals the common steps in azaphilone biosynthetic pathways to aid the use of genome mining to identify orphan azaphilone biosynthetic gene clusters.

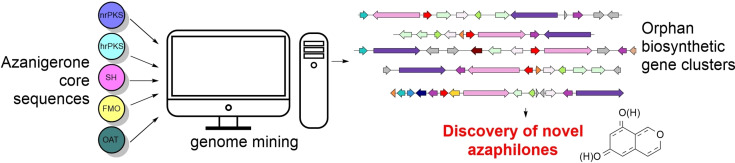

## 1 Introduction

**Figure 1:**
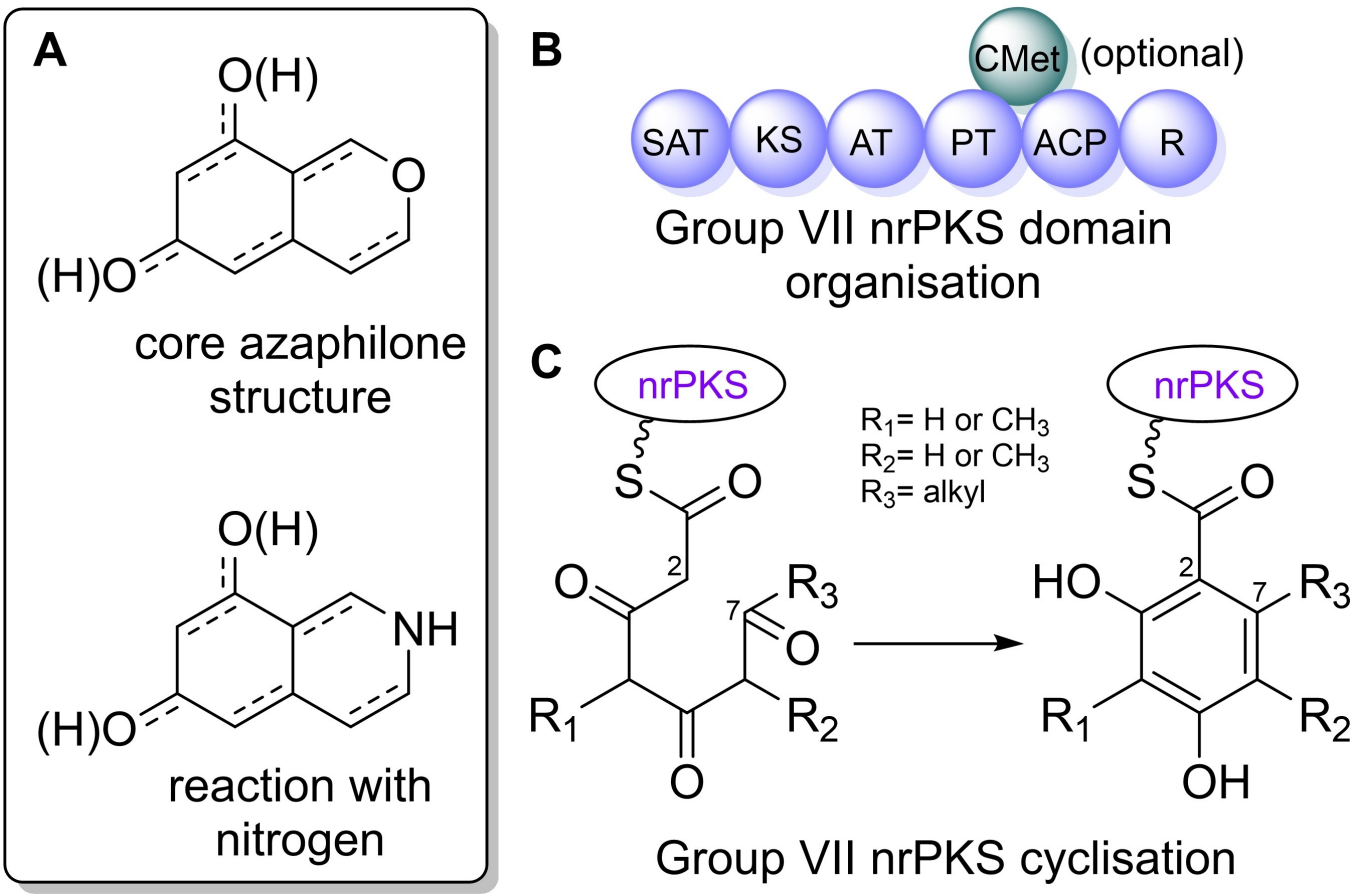
A) Core azaphilone structure, and reaction with nitrogen. B) Domain organisation of group VII nrPKSs. C) Cyclisation of the initial polyketide chain.

Azaphilones are a group of structurally related fungal natural products that contain a highly oxygenated bicyclic pyrone‐quinone structure, and a chiral quaternary centre. They can react readily with amines to form vinylogous γ‐pyridones, hence the name ‘azaphilone’ (Figure 1, A).[^1^, ^2^] Azaphilones exhibit a range of bioactivities including anti‐tumour, antifungal and antiviral activity, and are used as food colourants, and as dyes.^[1]^

This minireview focusses on the biosynthesis of azaphilone compounds and compares common biosynthetic steps between this family of natural products. We examine the biosynthetic pathways to several related compounds (where their biosynthesis informs studies on azaphilones): the important mycotoxin, citrinin **1**,^[3]^ and the anti‐cancer compound, asperfuranone **2**;^[4]^ as well as azaphilone biosynthetic pathways, such as to azanigerone A **3**.^[5]^ A consensus understanding of the core reactions that create the azaphilone structure, and the enzymes that catalyse them, can aid the use of genome mining to identify orphan azaphilone and azaphilone‐derived biosynthetic gene clusters (BGCs) for further investigation.

The azaphilone and related compounds discussed in this review are synthesised by group VII non‐reducing polyketide synthases (nrPKSs) (Figure S1).[^6^, ^7^] These nrPKSs all contain the following domains: SAT (starter unit‐ACP transacylase), KS (ketosynthase), AT (acyl transferase), PT (product template), ACP (acyl carrier protein), R (reductase), with some group VII nrPKSs also containing a CMeT (*C*‐methyltransferase) domain (Figure 1, B). The PT domain of group VII nrPKSs mediate a C‐2 to C‐7 cyclisation of the initial polyketide chain (Figure 1, C).^[7]^ The R domain cleaves the product *via* reductive release leaving an aldehyde group.^[6]^

## 2 Elucidation of Azaphilone and Relevant Related Biosynthetic Pathways

### 2.1 Citrinin (BGC0001338)

**Scheme 1:**
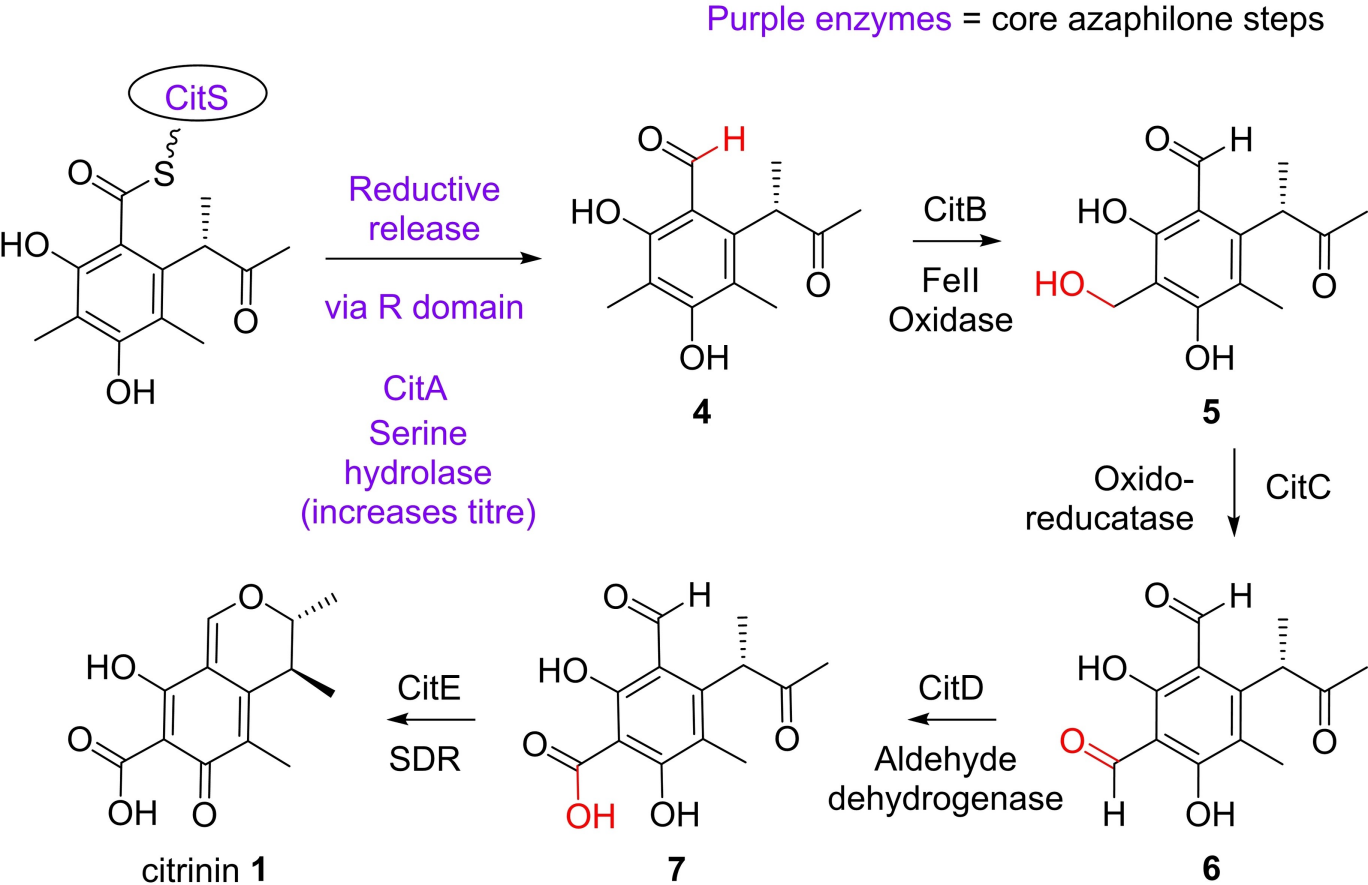
Proposed citrinin **1** biosynthetic pathway.

Citrinin **1** is a well‐known mycotoxin that is closely related to the azaphilones, but citrinin **1** itself is resistant to amination.^[3]^ Early investigations into citrinin **1** biosynthesis used radioisotopes, which confirmed its polyketide origin through the incorporation of ^14^C‐labelled acetate.[^8^, ^9^] Recent work has defined the molecular steps to citrinin **1** biosynthesis through a series of gene knockout and heterologous expression experiments (Scheme 1).^[3]^ Although citrinin **1** bears much structural similarity to azaphilones, consisting of a pyranoquinone bicyclic core, and is in fact often defined as an azaphilone,^[1]^ the molecular steps to its structure differentiate at an early stage. A key step, which is also present in some characterised azaphilone biosynthetic pathways, is the supportive role of the serine hydrolase (CitA in citrinin biosynthesis) to the nrPKS (CitS). He and Cox^[3]^ showed that it is required for high titre of the first enzyme free intermediate **4** by both knockout experiments in the producing strain, *Monascus ruber* M7, and heterologous expression experiments. Storm and Townsend^[10]^ have further clarified, but not completely elucidated the role of CitA: they show through *in vitro* assays that CitA can remove several enzyme‐bound acyl intermediates. They postulated that CitA functions as an *in trans* editing system that can remove acyl‐*holo*‐ACP species that are not direct intermediates to the programmed PKS product. Knowledge of this initial stage of citrinin biosynthesis informs our understanding of the equivalent steps found in most azaphilone‐based pathways. Further steps differ between citrinin and azaphilone biosynthesis.

### 2.2 Asperfuranone (BGC0000022)

**Figure 2:**
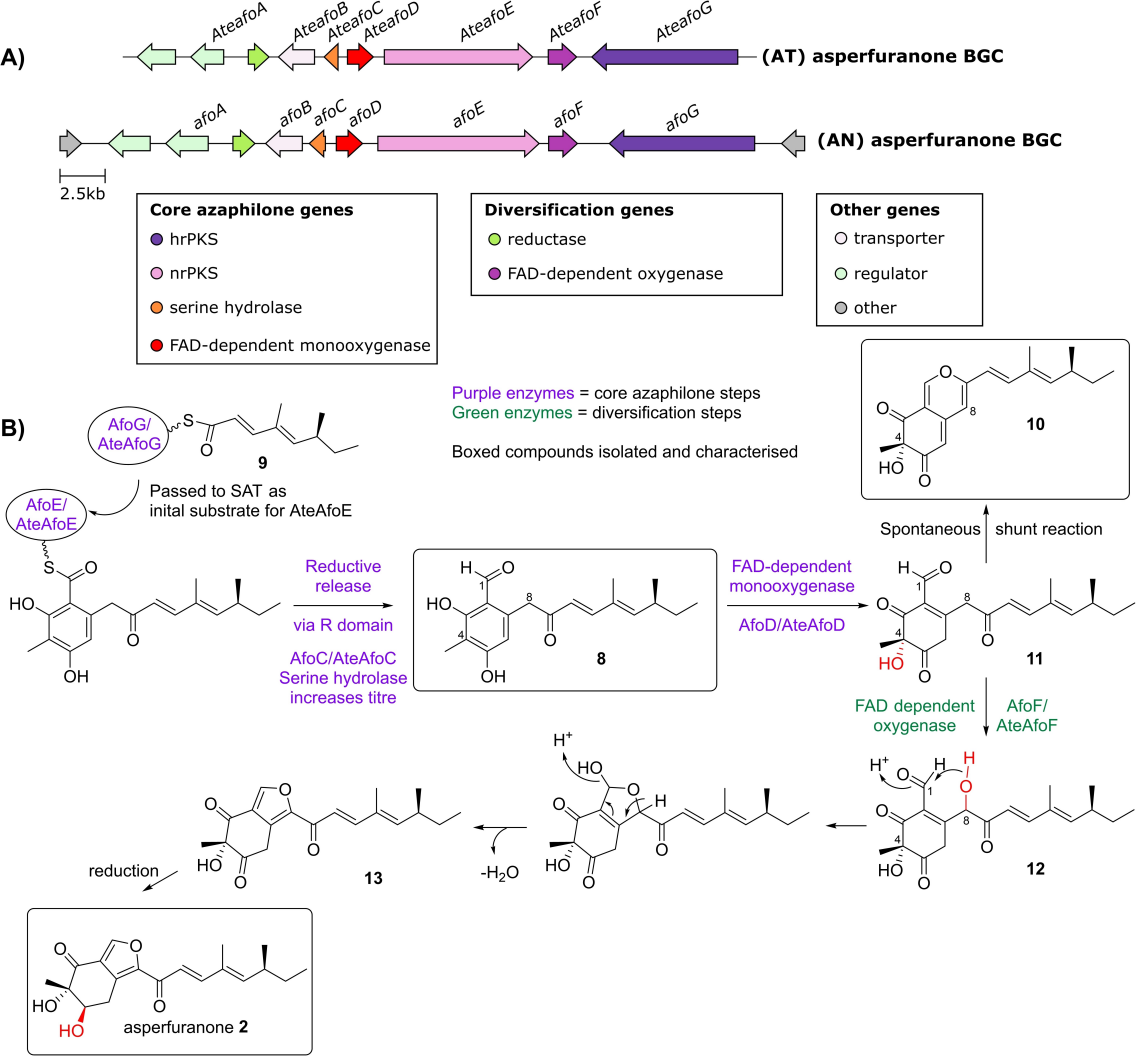
A) Asperfuranone BGCs from *Aspergillus terreus* (AT) and *Aspergillus nidulans* (AN), core genes required for azaphilone biosynthesis, as well as diversification genes are shown. B) Scheme showing proposed asperfuranone biosynthetic pathway.

Despite the multitude of known azaphilone compounds isolated from fungi, and extensive labelling studies to investigate azaphilone biosynthesis,[^1^, ^11^] until relatively recently little has been understood about the enzymatic steps, and the encoding genes. In 2009, Wang and co‐workers^[12]^ identified from the *Aspergillus nidulans* genome the biosynthetic gene cluster (BGC) responsible for the biosynthesis of asperfuranone **2**, a compound which is closely related to the azaphilones, consisting of a bicyclic furan containing core (Figure 2, A). This study provided the first hints towards the molecular pathway of the azaphilones, and showed that several of the enzymes are homologous to those involved in citrinin biosynthesis. Through gene deletion experiments, the authors showed that the serine hydrolase (AfoC) present in the BGC contributes to high titre of the first enzyme free intermediate **8** in a similar manner to citrinin **1** biosynthesis.[^3^, ^12^] The asperfuranone BGC contains both an nrPKS (*afoE*), homologous to *citS*, and a highly reducing PKS (hrPKS, *afoG*). Deletion of either PKS abolishes asperfuranone **2** biosynthesis, leading to the proposal that the hrPKS produces a reduced starter unit **9** that is transferred to the SAT domain of the nrPKS to be further extended.^[12]^ Further work in 2013 using an orthologous asperfuranone **2** BGC identified from *Aspergillus terreus* (Figure 2, A), completely reconstituted asperfuranone **2** biosynthesis in a heterologous host and demonstrated the order of each required step (Figure 2, B).^[13]^ Co‐expression of the hrPKS (AteAfoG) and the nrPKS (AteAfoE) led to isolation of compound **8** (Figure 2, B), demonstrating that both PKSs are required for biosynthesis of the first enzyme free intermediate. The addition of the serine hydrolase (AteAfoC) increased the yield of **8**. This pathway also revealed the essential azaphilone step is catalysed by an FAD‐dependent monooxygenase (FMO) with homology to salicylate monooxygenases. In the biosynthesis of asperfuranone, the FMO AteAfoD hydroxylates C‐4 of **8**, leading to spontaneous cyclisation and dehydration to give the shunt azaphilone **10** (Figure 2, B), with the characteristic bicyclic pyrone‐quinone structure. The mature compound, asperfuranone **2**, is formed after C‐8 hydroxylation of intermediate **11**, by an FAD‐dependent oxygenase, AteAfoF, giving **12**. Cyclisation and dehydration gives the five membered furan **13** and reduction leads to asperfuranone **2** (Figure 2, B).^[13]^

### 2.3 Azanigerone A (BGC0001143)

**Figure 3:**
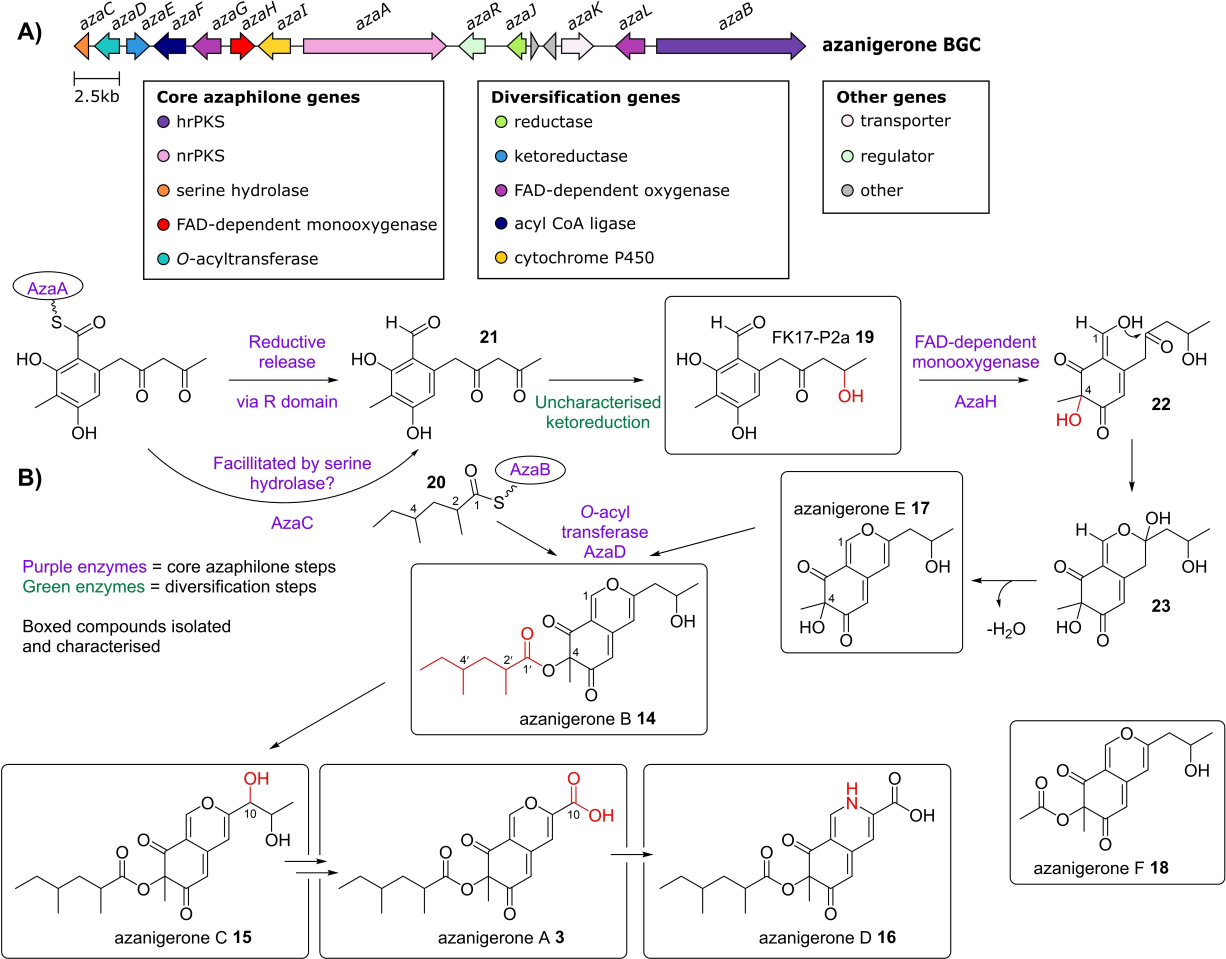
A) Azanigerone BGC, core genes required for azaphilone biosynthesis, as well as diversification genes are shown. B) Scheme showing proposed azanigerone biosynthetic pathway.

The first BGC encoding azaphilone biosynthesis was reported by Tang and co‐workers in 2012.^[5]^ A BGC was identified within the *Aspergillus niger* genome which contained both an nrPKS (*azaA*) and a hrPKS (*azaB*) (Figure 3, A), which was minimally expressed under conditions screened.^[5]^ Comparison with the asperfuranone **2** cluster revealed homologues between seven of the catalytic protein sequences from the *aza* BGC, suggesting that the cluster might code for the production of an asperfuranone‐like compound. No candidate compounds had previously been isolated from *A. niger*, therefore overexpression of the putative transcription factor present within the BGC was performed. This led to the production and isolation of seven compounds, six of which were novel azaphilones, azanigerone A **3** and the azanigerones B−F, **14**–**18**.^[5]^ The seventh compound was the tetrasubstituted benzaldehyde FK17‐P2a **19** which has previously been isolated from other fungi.[^14^, ^15^] *In vitro* characterisation of the FAD‐dependent monooxygenase (AzaH, a homologue of AteAfoD) demonstrated that is solely responsible for the flavin‐dependent conversion of the benzaldehyde intermediate FK17‐P2a **19** into the bicyclic, pyran‐containing azaphilone compound, azanigerone E **17** (Figure 3, B). Deletion of the hrPKS (*azaB*), in contrast to the asperfuranone pathway, led to the accumulation of azanigerone E **17**. This suggested that AzaB is only responsible for the biosynthesis of the 2,4‐dimethylhexanoyl chain **20**, which is proposed to be the substrate for the *O‐*acyltransferase AzaD to esterify the C‐4 hydroxyl group of azanigerone E **17** to produce azanigerone B **14**. Additional predictions were made about the pathway, based on a time course experiment and isolation of the metabolites, but not further verified.^[5]^

### 2.4 Chaetoviridins and chaetomugilins (BGC0001405)

**Figure 4:**
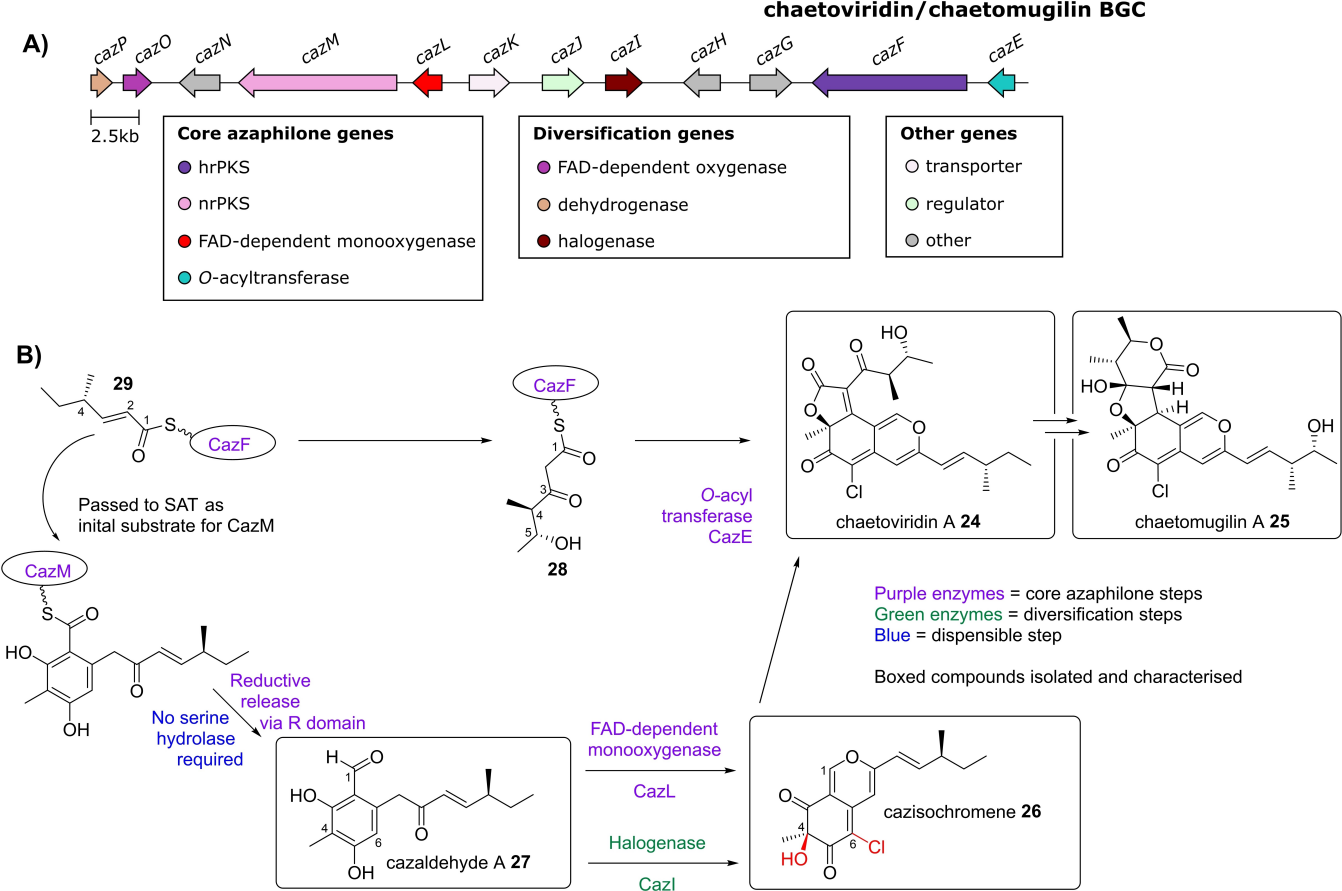
A) Chaetoviridins and chaetomugilins BGC, core genes required for azaphilone biosynthesis, as well as diversification genes are shown. B) Scheme showing proposed chaetoviridin A biosynthetic pathway. Carbons are numbered to correspond to azanigerone A **3**.

Characterisation of a second azaphilone pathway was reported in 2012 by Tang and co‐workers,^[16]^ following studies on the biosynthesis of chlorinated azaphilone compounds produced by *Chaetomium globosum*, for example chaetoviridin A **24** ^[17]^ and chaetomugilin A **25**.^[18]^ The BGC that codes for production of these compounds was identified from the genome of *C. globosum* by searching for clustered homologues of the nrPKSs and hrPKSs present in the asperfuranone and azanigerone BGCs (Figure 4, A). Knock out of either the nrPKS (*cazM*) or the hrPKS (*cazF*) abolished production of any azaphilone compounds, with no other intermediates accumulating. Knock‐out of the *O*‐acyltransferase, *cazE* (which has a homologue in the azanigerone A **3** BGC, *azaD*) accumulated cazisochromene **26** (Figure 4, B), which is structurally related to azanigerone E **17** (an intermediate in the azanigerone A **3** pathway). Cazisochromene **26** is likely to be the product of C‐4 hydroxylation by CazL and C‐6 chlorination by CazI of cazaldehyde A **27**. Timing of chlorination versus hydroxylation is unclear. Interestingly, no serine hydrolase is present in this BGC, suggesting that its activity is not always essential in azaphilone biosynthesis. *In vitro* assays using CazF (hrPKS), CazE (*O*‐acyltransferase), and cazisochromene **26**, resulted in the production of chaetoviridin A **24**. This is in accord with the hrPKS giving the 5‐hydroxy‐4‐methyl‐3‐oxohexanoyltriketide **28** that is required to produce the lactone ring fused to the isochromenone core of chaetoviridin A **24**. The lack of any azaphilone compounds produced by the Δ*cazF* strain suggested that the hrPKS is also implicated in the production of the 4‐methyl‐hex‐2‐enoyltriketide starter unit **29** required by the nrPKS (CazM).^[16]^ This was confirmed in a later study,^[19]^ whereby *in vitro* assays of CazF and CazM produced cazaldehyde A **27**, the first enzyme free intermediate that undergoes hydroxylation and chlorination before cyclisation and dehydration forms the azaphilone core. Experiments showed that the SAT domain only recognised the more reduced triketide.^[19]^ This is an interesting example of an hrPKS producing two differing substrates required for the biosynthesis of the mature compound.

### 2.5 *Monascus* spp. azaphilones (BGC0000027)

**Figure 5:**
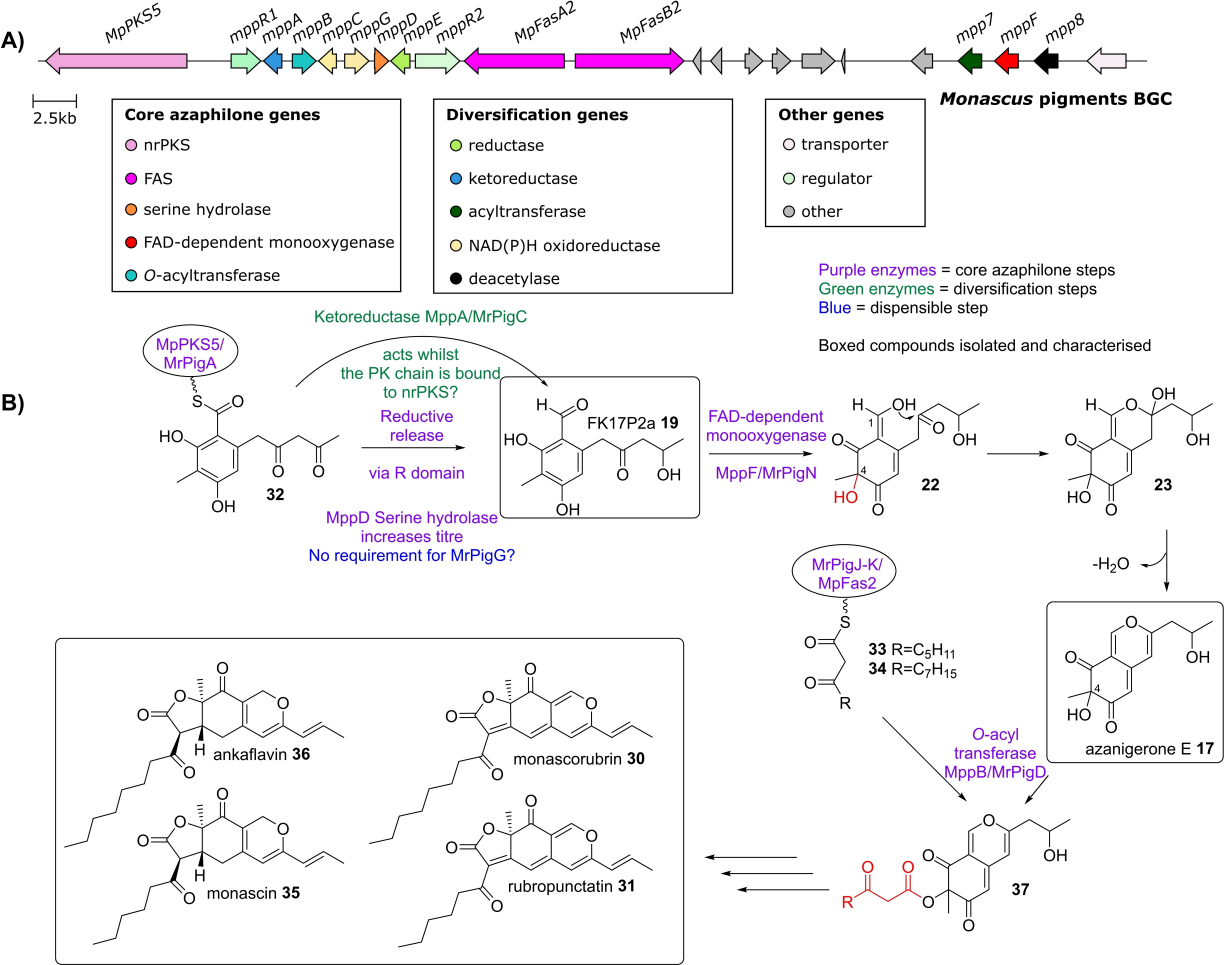
A) *Monascus* pigments BGC, core genes required for azaphilone biosynthesis, as well as diversification genes are shown. B) Scheme showing proposed *Monascus* spp. azaphilone biosynthetic pathway.

The BGC for the well‐known food colourant azaphilones isolated from *Monascus* spp. such as monascorubrin **30** and rubropunctatin **31** was reported in 2013 by Kwon and co‐workers (Figure 5, A).^[20]^ This was achieved by random T‐DNA mutagenesis of *Monascus purpureus* and screening for pigment loss. At the time, the genome of *M. purpureus* had not been sequenced, therefore the available *Monascus pilosus* genome was used to align the sequence of the T‐DNA insertion region to identify a putatively orthologous BGC. Once identified, the nrPKS present in the cluster (*MpPKS5*), which is homologous to other known azaphilone nrPKSs, was targeted for gene deletion in *M. purpureus*, to create strain Δ*MpPKS5*. This strain no longer produced any azaphilones, demonstrating that MpPKS5 is essential to azaphilone biosynthesis in *M. purpureus*. Kwon and co‐workers^[21]^ later showed that the serine hydrolase (MppD) present in the BGC contributes to high titre of the first enzyme free intermediate **19** in a similar manner to citrinin **1** and asperfuranone **2** biosynthesis.[^3^, ^12^, ^21^] Interestingly, an orthologous cluster from *Monascus ruber* studied by Chen and co‐workers^[22]^ did not appear to possess the same reliance on the serine hydrolase for high titre. Co‐expression of the nrPKS (MrPigA) with the serine hydrolase (MrPigG) in the heterologous host *Saccharomyces cerevisiae* did not increase yield compared to expression of MrPigA alone.^[22]^ Studies by both groups show that the orthologs MppA and MrPigC catalysed the ketoreduction of the highly reactive benzaldehyde **32** to give **19**.[^22^, ^23^] There is no homologue to MppA/MrPigC in the azanigerone A **3** cluster, although the same ketoreduction does occur, and Tang and co‐workers speculated that AzaE is instead responsible.^[5]^ The FAD‐dependent monooxygenase orthologues MppF/MrPigN catalyse the C‐4 ring hydroxylation of FK17‐P2a **19** to form azanigerone E **17** (Figure 5, B).[^22^, ^23^] In these BGCs a fatty acid synthase dimer, MpFas2/MrPigJ‐K catalyses the biosynthesis of the fatty acid moieties **33** and **34**, which are present in the *Monascus* spp. azaphilones, unlike the hrPKSs that are present in the azanigerone A **3** or the chaetoviridin/chaetomugilins BGCs.[^22^, ^24^] An *O*‐acyltransferase, MrPigD, homologous to those found in the azanigerone A **3** and the chaetoviridin/chaetomugilins BGCs, transfers the fatty acid moieties to the C‐4 hydroxyl group. This was demonstrated by the creation of a Δ*MrPigD* strain which accumulates azanigerone E **17**.^[22]^ The *O‐*acyltransferase homologue MppA from the *Monascus purpureus* BGC is proposed to perform the same reaction.^[24]^ Further steps lead to generation of monascin **35**, rubropunctatin **31**, ankaflavin **36** and monascorubrin **30**.[^22^, ^25^, ^26^, ^27^]

### 2.6 Trigazaphilones (LVVK01000021.1, 680,000‐727,296)

**Figure 6:**
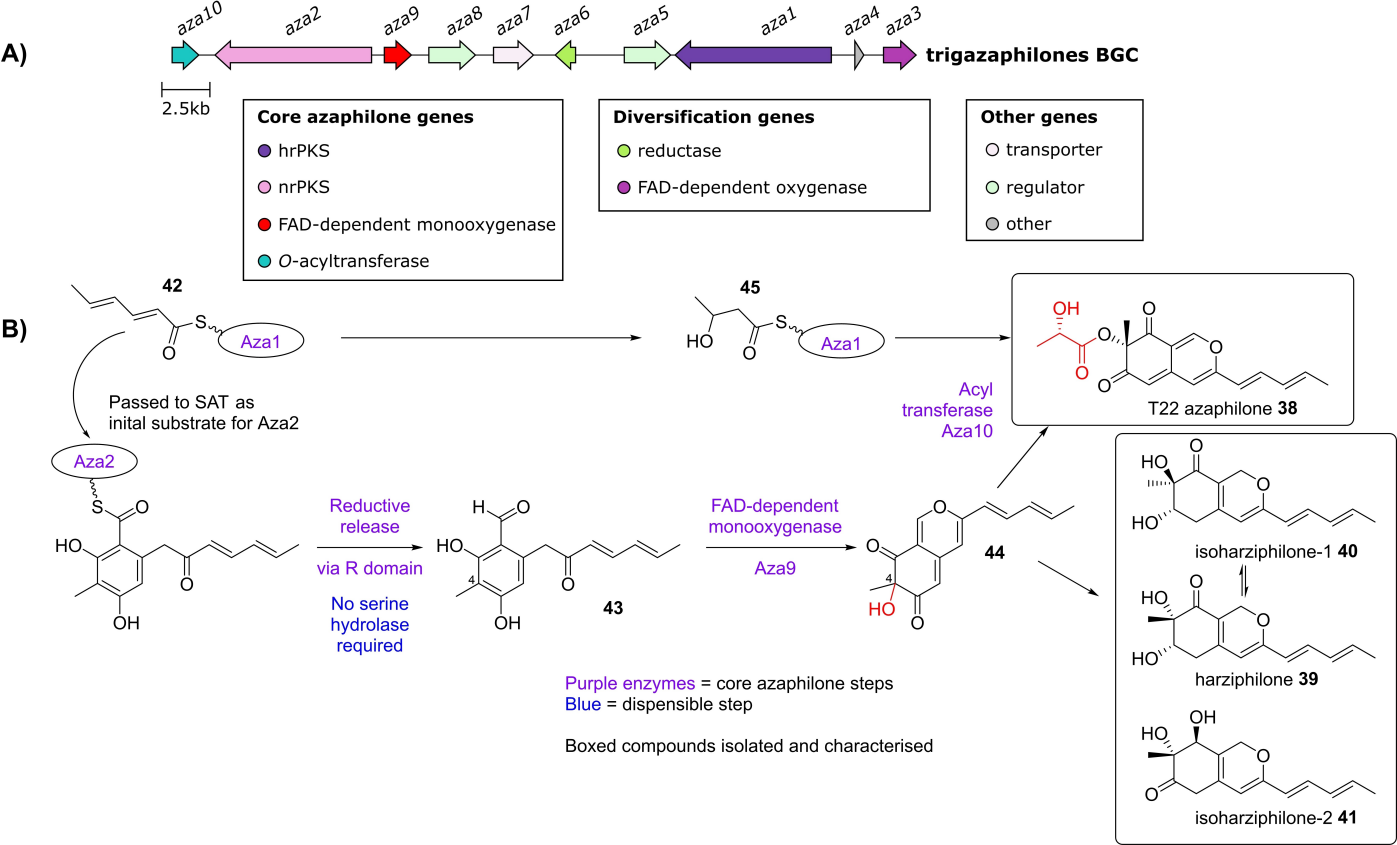
A) Trigazaphilones BGC, core genes required for azaphilone biosynthesis, as well as diversification genes are shown. B) Scheme showing proposed trigazaphilones biosynthetic pathway.

Recently, a BGC identified from the mycoparasitic fungus *Trichoderma guizhouense* was shown to encode the biosynthesis of azaphilones. Zhang and co‐workers^[28]^ noticed the production of a ‘light yellow pigment’ when *T. guizhouense* interacts with and inhibits the growth of *Fusarium odoratissimum*. Through analysis of a transcriptome profile of this interaction,^[29]^ upregulated BGCs were identified, one of which was shown to be responsible for the biosynthesis of the ‘light yellow pigment’, through deletion of the two PKS genes encoded within the BGC (*aza1* and *aza2*, Figure 6, A). The compounds were isolated and characterised as T22 azaphilone **38**, harziphilone **39**, isoharziphilone‐1 **40** and isoharziphilone‐2 **41**, which collectively have been named the trigazaphilones (Figure 6, B). Deletion of the *O‐*acyltransferase encoded with the BGC (*aza10*) abolished T22 azaphilone **38** biosynthesis, with the deletion strain accumulating harziphilone **39** and its isomers **40** and **41**. The authors proposed that the biosynthesis proceeds in a similar manner to that of the chaetoviridins and chaetomugilins (Figure 6, B). Interestingly, this BGC represents another azaphilone biosynthetic pathway which has no serine hydrolase, supporting the dispensable nature of this enzyme.

## 3 Consensus Pathway

**Scheme 2:**
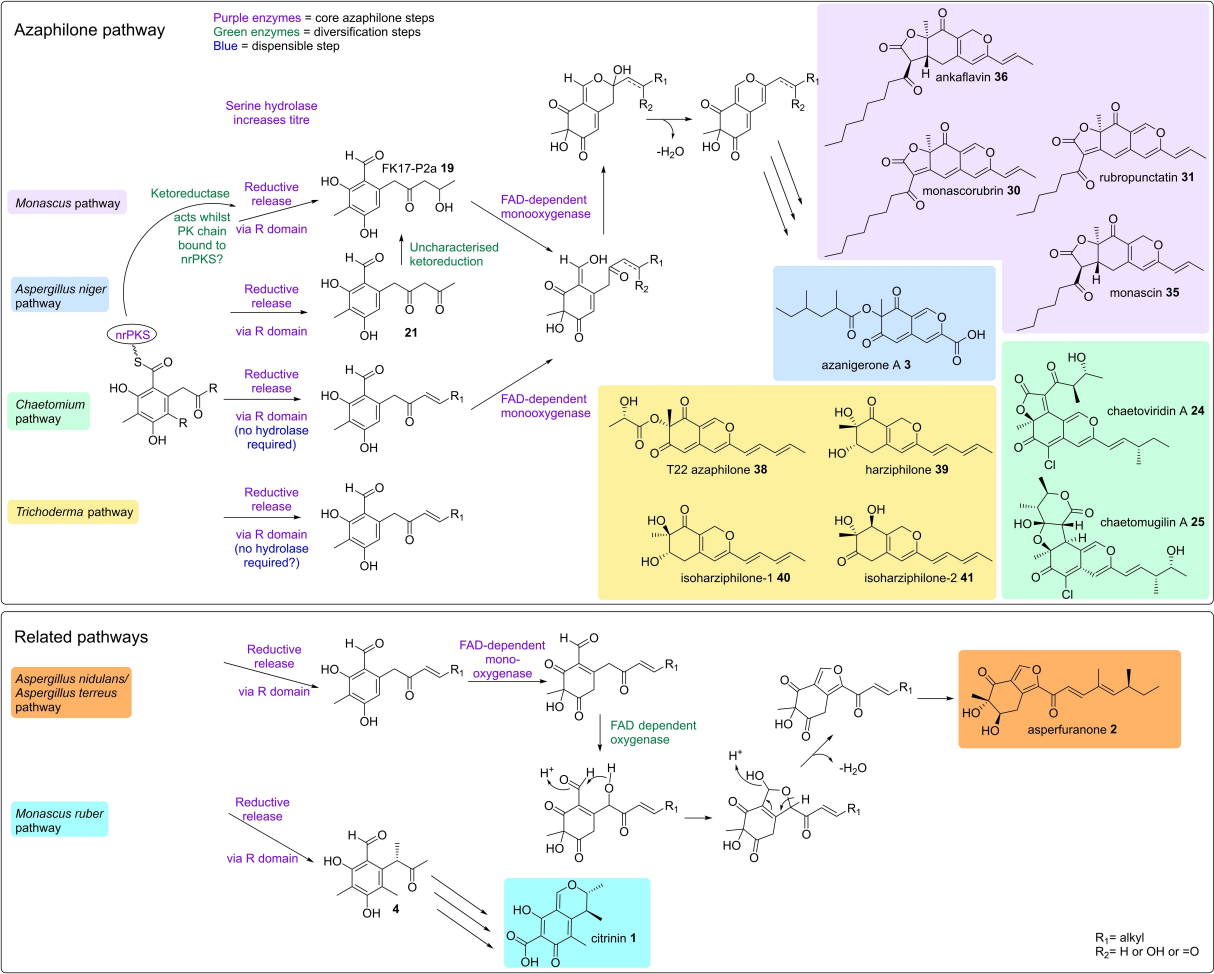
A comparison of azaphilone and several related pathways.

The current consensus (Scheme 2) for the core steps required for azaphilone biosynthesis are exemplified by the azanigerone A **3** pathway. In fact, azanigerone E **17**, an intermediate in azanigerone A **3** biosynthesis (Figure 3, B) is also an intermediate in *Monascus* spp. pigment biosynthesis (Figure 5, B), furthermore, the structurally related cazisochromene **26**, is an intermediate in the biosynthesis of chaetoviridins and chaetomugilins (Figure 4, B), as well as a similar analogue (**10**) being produced as a shunt compound during asperfuranone **2** biosynthesis (Figure 2, B). Azanigerone E **17** (or analogues) require an nrPKS to biosynthesise a benzaldehyde intermediate, the titre of which may be increased by the serine hydrolase (although it is not always necessary). The nrPKS may require an hrPKS to produce a specific reduced starter unit for further extension.

**Figure 7:**
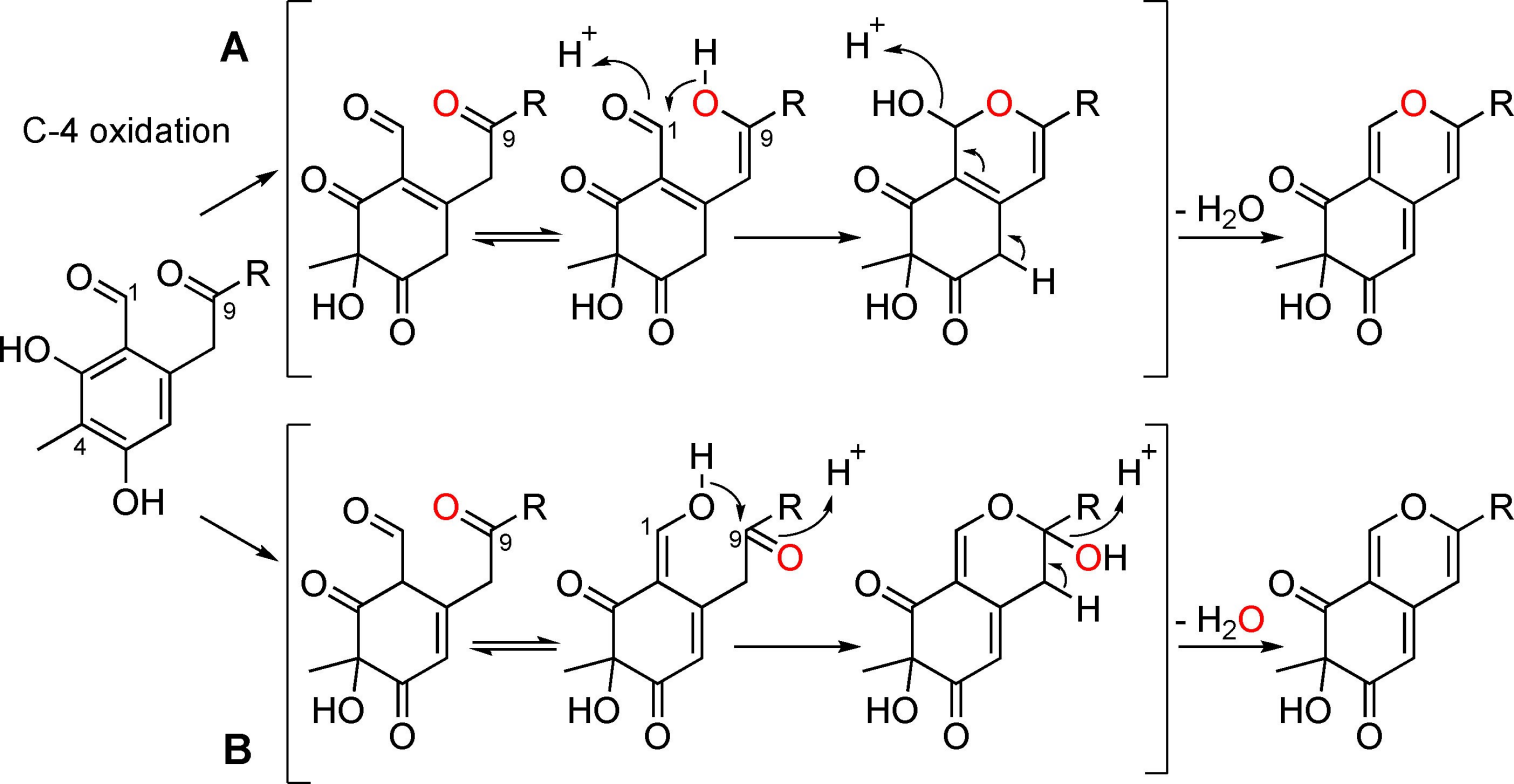
Differing azaphilone bicyclic ring formation mechanisms. Mechanism A was proposed by Wang and co‐workers,^[13]^ mechanism B was proposed by Tang and co‐workers.^[5]^ A labelling study (labelled oxygen depicted in red) would distinguish the correct mechanism.

The FAD‐dependent monooxygenase catalyses C‐4 hydroxylation, which is the key step in azaphilone biosynthesis, converting the benzaldehyde into the bicyclic, pyran‐containing core of azaphilones. It is interesting to note that two mechanisms have been suggested for the biosynthesis of this core structure. Wang and co‐workers^[13]^ proposed that the C‐1 aldehyde group acts as an electrophile, whilst Tang and co‐workers^[5]^ proposed that the same group acts as a nucleophile. Labelling studies could distinguish the correct mechanism (Figure 7).

The C‐4 hydroxyl group introduced by the FAD‐dependent monooxygenase is then a target for a further common step in azaphilone biosynthesis, acylation of the oxygen by an *O*‐acyltransferase. The acyl moiety can be biosynthesised by either an hrPKS (which can be the same hrPKS that produces the starter unit, as seen in Figure 4, B), or an FAS, as seen *Monascus* spp. pigment biosynthesis (Figure 5, B). Diversification of azaphilone structures can occur in these early stages, for example, the requirement for a starter unit for the nrPKS, which can vary in length and reduction, or the halogenation that occurs in chaetoviridin and chaetomugilin pathway (Figure 4, B). Further diversification can also occur after the transfer of the acyl moiety, to produce the range of known azaphilone structures, some of which are illustrated in Scheme 2, as well as other more complex azaphilone‐based compounds, for example, the dimeric azaphilones.^[30]^ A recent review by Franck and co‐workers discusses various azaphilone diversification steps.^[31]^

**Figure 8:**
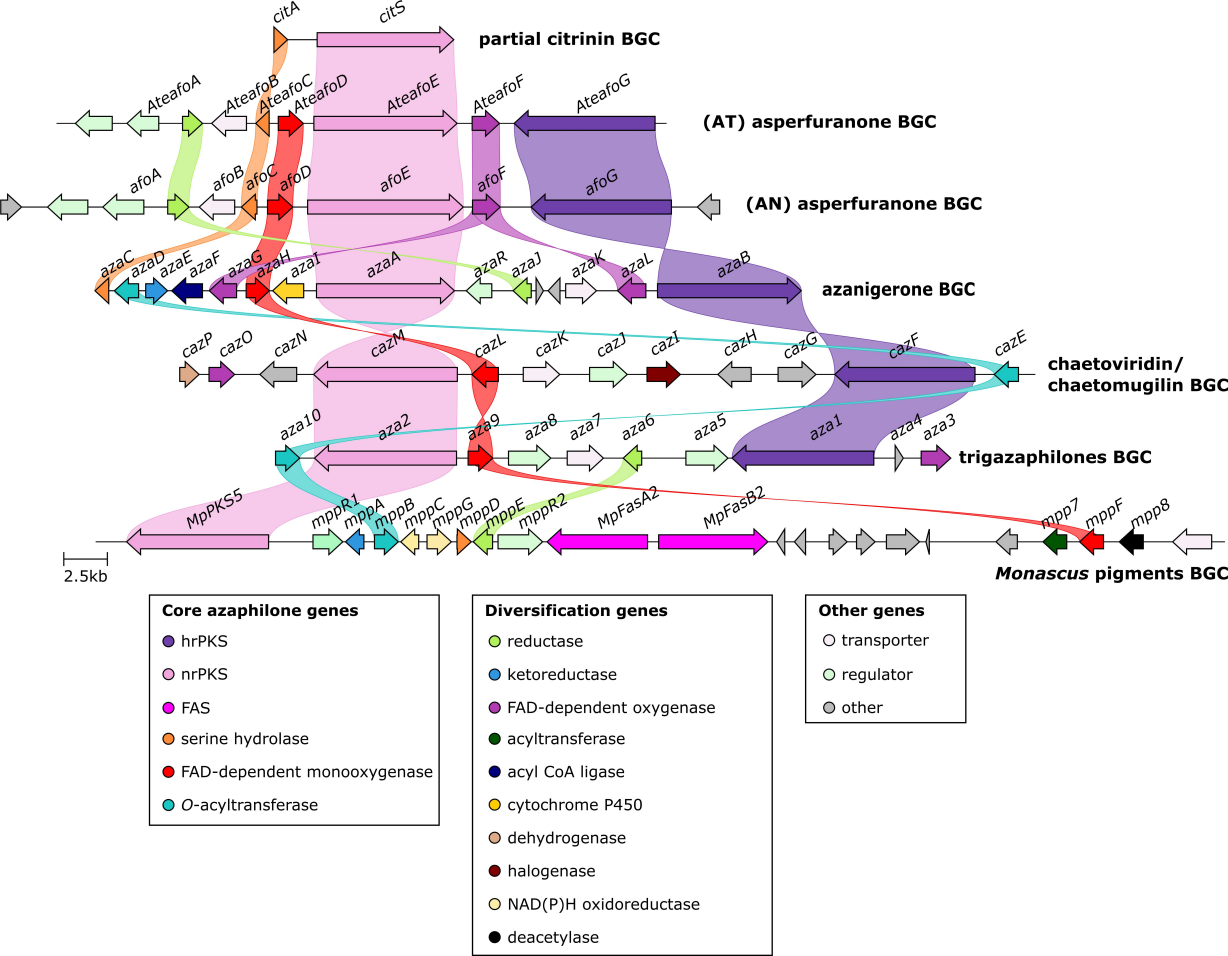
Clinker^[32]^ comparison of the azaphilone and related BGCs described above. For the citrinin BGC, only the relevant azaphilone related genes are shown. The *Monascus* pigments *Monascus ruber* BGC genomic sequence was not publicly available for comparison. The asperfuranone sequences are from *Aspergillus nidulans* (AN) and *Aspergillus terreus* (AT).

A clinker^[32]^ comparative analysis of the BGCs discussed in sections 2.1–2.6 (Figure 8) shows the similarities and differences between these clusters. It is clear that each gene is not always necessary for azaphilone biosynthesis, but we hypothesise that their clustered presence within a fungal genome would be highly indicative of a BGC that codes for the production of an azaphilone compound. Core protein sequences that can be used for identifying further azaphilone BGCs from genome sequences are AzaA (nrPKS), AzaB (hrPKS), AzaC, a serine hydrolase (SH), AzaH, an FAD‐dependent monooxygenase (FMO) and AzaD, an *O*‐acyltransferase (OAT) (Table 1).

**Table 1:** Percentage identity between core protein sequences from azaphilone and related pathways compared to the azanigerone core sequences.^[a]^ Accession numbers for each protein are shown underneath the protein name. The citrinin sequences are from *Monascus ruber* (MR). The asperfuranone sequences are from either *Aspergillus nidulans* (AN) or *Aspergillus terreus* (AT). The *Monascus* pigment sequences are from either *Monascus pilosus* (MPi) or from *Monascus ruber* (MR).

Function	Azanigerone	% Identity compared to azanigerone core protein sequences						
		Citrinin (MR)	Asperfuranone (AN)	Asperfuranone (AT)	*Monascus* pigments (MPi)	*Monascus* pigments (MR)	Chaetoviridin	Trigazaphilones
nrPKS	**AzaA G3XMC4.1**	CitS (47.87) **A0A161CEU9.1**	AfoE (45.66) **Q5BEJ6.1**	AteAfoE (46.39) **Q0CF73.1**	MpPKS5 (58.12) **AGN71604.1**	MrPigA (58.23) **ALN44200.1**	CazM (46.05) **AKA40070.1**	Aza2 (46.64) **OPB37950.1**
hrPKS	**AzaB G3XMD1.1**	n/a	AfoG (47.14) **Q5BEJ4.1**	AteAfoG (47.54) **Q0CF75.1**	n/a	n/a	CazF (47.95) **EAQ86385.1**	Aza1 (44.82) **OPB37944.1**
SH	**AzaC G3XMB7.1**	CitA (47.28) **A0A161CKG1.1**	AfoC (48.16) **Q5BEJ8.1**	AteAfoC (51.26) **Q0CF71.1**	MppD^[b]^ (57.32)	MrPigG (58.54) **APZ73942.1**	n/a	n/a
FMO	**AzaH G3XMC2.1**	n/a	AfoD (41.05) **Q5BEJ7.1**	AteAfoD (41.63) **Q0CF72.1**	MppF (58.27) **AGN71623.1**	MrPigN (59.23) **ALT31754.1**	CazL (42.08) **EAQ86391.1**	Aza9 (37.68) **OPB37949.1**
OAT	**AzaD G3XMB8.1**	n/a	n/a	n/a	MppB (49.22) **AGN71607.1**	MrPigD (49.22) **AGI63864.1**	CazE (33.79) **EAQ86384.1**	Aza10 (33.04) **OPB37951.1**
FAS‐α	**n/a**	n/a	n/a	n/a	MpFasA2 **AGN71613.1**	MrPigJ **AGL44429.1**	n/a	n/a
FAS‐β	**n/a**	n/a	n/a	n/a	MpFasB2 **AGN71614.1**	MrPigK **AGL44430.1**	n/a	n/a

[a] nrPKS=non‐reducing polyketide synthase, hrPKS=highly reducing polyketide synthase, SH=serine hydrolase, FMO=FAD‐dependent monooxygenase, OAT=*O*‐acyltransferase, FAS‐α/β=fatty acid synthase subunit. n/a=not applicable, as no homologue within the BGC. [b] MppD was originally annotated incorrectly, and the sequence was later revised, but the revised sequence is not publicly available, therefore the sequence was manually derived *via* homology searches (see Figure S2).^[25]^

To test this theory, we have conducted a representative cblaster^[33]^ search for co‐localised genes from annotated genomes on the NCBI database, using the above five sequences as queries, with AzaA, AzaD and AzaH set as required (Figure S3). This search detected 193 BGCs with azaphilone producing potential, 8 of which have been either definitively[^5^, ^28^, ^34^, ^35^, ^36^] or putatively[^30^, ^37^] linked to the biosynthesis of azaphilone derived compounds. This leaves 185 orphan BGCs that do not appear yet to be linked to specific compound biosynthesis, however many of the species have known azaphilone producers within their genera.[^2^, ^11^, ^31^] Orphan BGCs are candidates for further investigation into azaphilone biosynthesis and production. Other cblaster^[33]^ searches could be attempted, for example, without AzaD set as required (as the *O*‐acylation step is not necessary for the formation of the bicyclic pyrone‐quinone azaphilone core), or alternatively using the MpFas2/MrPigJ‐K fatty acid synthase dimer sequences instead of AzaB, or using diversifying enzymes sequences, for example CazI, the halogenase from the chaetoviridins and chaetomugilins BGC.

## 4 Summary and Outlook

Fungal genome mining is an exciting method to help realise the full natural product potential revealed within fungal genomes. Many tools exist to facilitate fungal genome screening, two of the most popular are fungiSMASH^[38]^ and SMURF,^[39]^ both of which use profile Hidden Markov Model (pHMM) based algorithms to identify BGCs, along with other complementary algorithms such as CASSIS (uses co‐regulatory motifs),^[40]^ CO‐OCCUR (shared syntenic relationships among genes)^[41]^ and FunOrder^[42]^ (co‐evolutionary linked genes). An understanding of the common biosynthetic steps towards a particular family of compounds can help identify specific BGCs amongst the wealth of data that the number of sequenced fungal genomes provides. Co‐localisation of homologues to specific sequences from the azanigerone BGC, (AzaA (nrPKS), AzaB (hrPKS), AzaC (serine hydrolase), AzaH (FAD‐dependent monooxygenase) and AzaD, (*O*‐acyltransferase) see Table 1) is highly likely to indicate the presence of an azaphilone BGC within a fungal genome. Many of the species identified by the cblaster^[33]^ analysis shown in Figure S3 are not specifically known to produce azaphilones.[^2^, ^11^, ^31^] Investigation of orphan azaphilone BGCs, especially in species where azaphilones have not previously been detected (and therefore might indicate unexplored chemical space) could be a fruitful avenue for the discovery and isolation of novel azaphilones.

## Conflict of interest

The authors declare no conflict of interest.

## Biographical Information

*Dr. Katherine Williams received her PhD from the University of Bristol (UoB) in 2010, before moving to the Bristol Polyketide Group, investigating the biosynthesis of fungal metabolites with interesting bioactive properties. Research posts at the Leibniz Universität Hannover, Germany with Prof. Russell Cox, and at Cardiff University with Prof. Ruedi Allemann followed. She now works in the UoB, with Dr. Andy Bailey and Prof. Chris Willis, on a project aimed at developing a high‐throughput heterologous production platform for fungal natural product antibiotic discovery*.

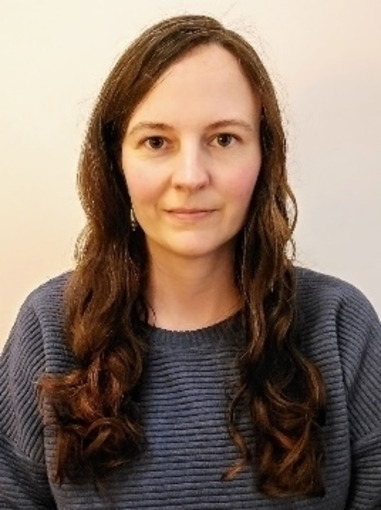

## Biographical Information

*Dr. Claudio Greco received his PhD at the University of Bristol (2017) elucidating the biosynthesis of fungal secondary metabolites under the supervision of Prof. Russell Cox and Prof. Chris Willis. This was followed by a two‐year postdoctoral research at the University of Wisconsin‐Madison working with Prof. Nancy Keller, studying secondary metabolism regulation in pathogenic fungi. He is currently working at the John Innes Centre with Prof. Barrie Wilkinson as a BBSRC Discovery Fellow investigating the ecological role of fungal natural products*.

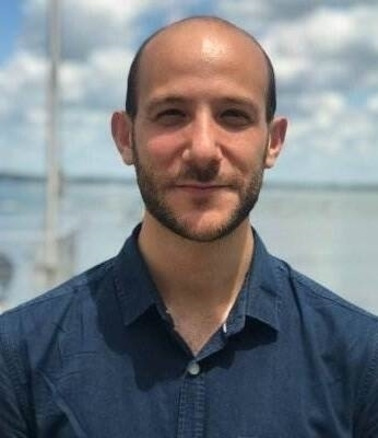

## Biographical Information

*Dr. Andy Bailey is a senior lecturer at the University of Bristol, UK. His research interests are based around different aspects of fungal biology and their analysis using molecular genetic approaches. This includes genome mining to explore fungal secondary metabolism, fungi as pathogens of plants and invertebrates and other fungi, plus establishing methods for genetic analysis of basidiomycetes*.

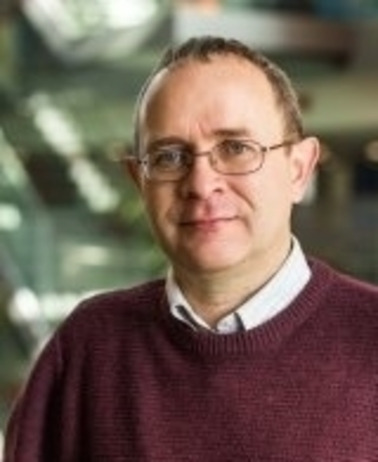

## Biographical Information

*Prof. Chris Willis is Professor of Organic Chemistry and Head of Organic and Biological Chemistry at the University of Bristol. Her research focuses on natural product biosynthesis including the application of total synthesis, isotopic labelling, pathway engineering and mechanistic studies to produce biocatalysts and new bioactive molecules. She was the recipient of the Natural Product Chemistry Award of the Royal Society of Chemistry in 2020*.

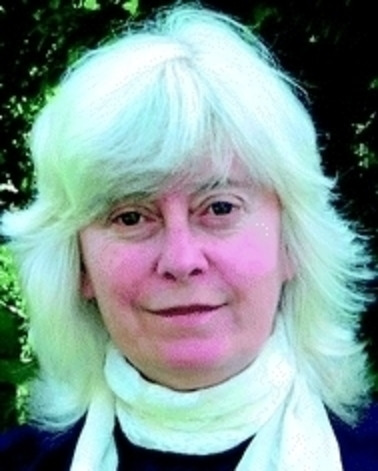

## Supporting information

As a service to our authors and readers, this journal provides supporting information supplied by the authors. Such materials are peer reviewed and may be re‐organized for online delivery, but are not copy‐edited or typeset. Technical support issues arising from supporting information (other than missing files) should be addressed to the authors.

Supporting Information
