# Supplementary material for: Core Steps to the Azaphilone Family of Fungal Natural Products
Source: Chembiochem. 2021 Jul 9;22(21):3027–36. doi: 10.1002/cbic.202100240 (PMC8596599; doi:10.1002/cbic.202100240)
Supplement: Supplementary file 1 — Supporting Information [file CBIC-22-3027-s001.pdf]

ChemBioChem

Supporting Information

Core Steps to the Azaphilone Family of Fungal Natural Products

Katherine Williams,* Claudio Greco, Andrew M. Bailey, and Christine L. Willis

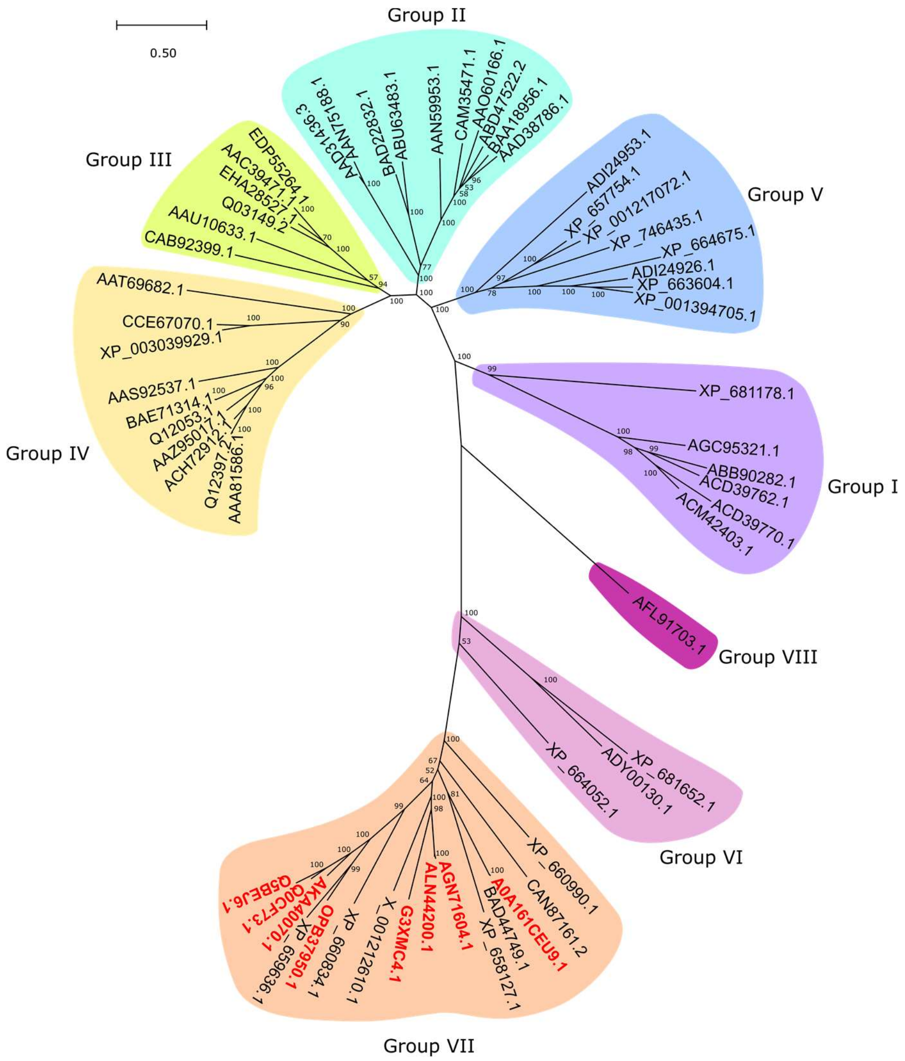

Figure S1: Phylogenetic analysis of fungal nrPKS sequences using data from Wang and co-workers.[1] Sequences discussed in the manuscript are depicted in red, and all fall within the Group VII clade. The evolutionary history was inferred by using the Maximum Likelihood method and Le_Gascuel_2008 model.[2] The tree with the highest log likelihood (-128284.24) is shown. Initial tree(s) for the heuristic search were obtained automatically by applying Neighbour-Join and BioNJ algorithms to a matrix of pairwise distances estimated using a JTT model, and then selecting the topology with superior log likelihood value. A discrete Gamma distribution was used to model evolutionary rate differences among sites (5 categories (+G, parameter = 1.2893)). The rate variation model allowed for some sites to be evolutionarily invariable ([+I], 1.61% sites). The tree is drawn to scale, with branch lengths measured in the number of substitutions per site. This analysis involved 60 amino acid sequences. All positions with less than 95% site coverage were eliminated, i.e., fewer than 5% alignment gaps, missing data, and ambiguous bases were allowed at any position (partial deletion option). There were a total of 1583 positions in the final dataset. Evolutionary analyses were conducted in MEGA X.[3]

```
>MppD_deduced_sequence
MPAKPSSDDSTLHLPRILCLHGGGTNARIFRAQCRILTRHFSTAFRLCFAEAPFVSQPGP
DVTSVYKEFGPFKRWLRSSARDHPEIDPALATEAIDASLRDAMDEDDRRGATGEWAGLLG
FSQGAKMCASLLLRQQVRDSTRTRPSATDGPHWRFAVLLAGRGPLVSLDPACPKSPAIVD
AGGIAMTAFPDERLLAAGTAHVLHLPTVHVHGMLDPGLEEHRKLLTQYCAEGTARVMEWE
GNHRVPIKTKDVVALAGHVFDVALETGVLQRRT
```

Figure S2: Sequence of MppD, which has been manually derived *via* homology searches.

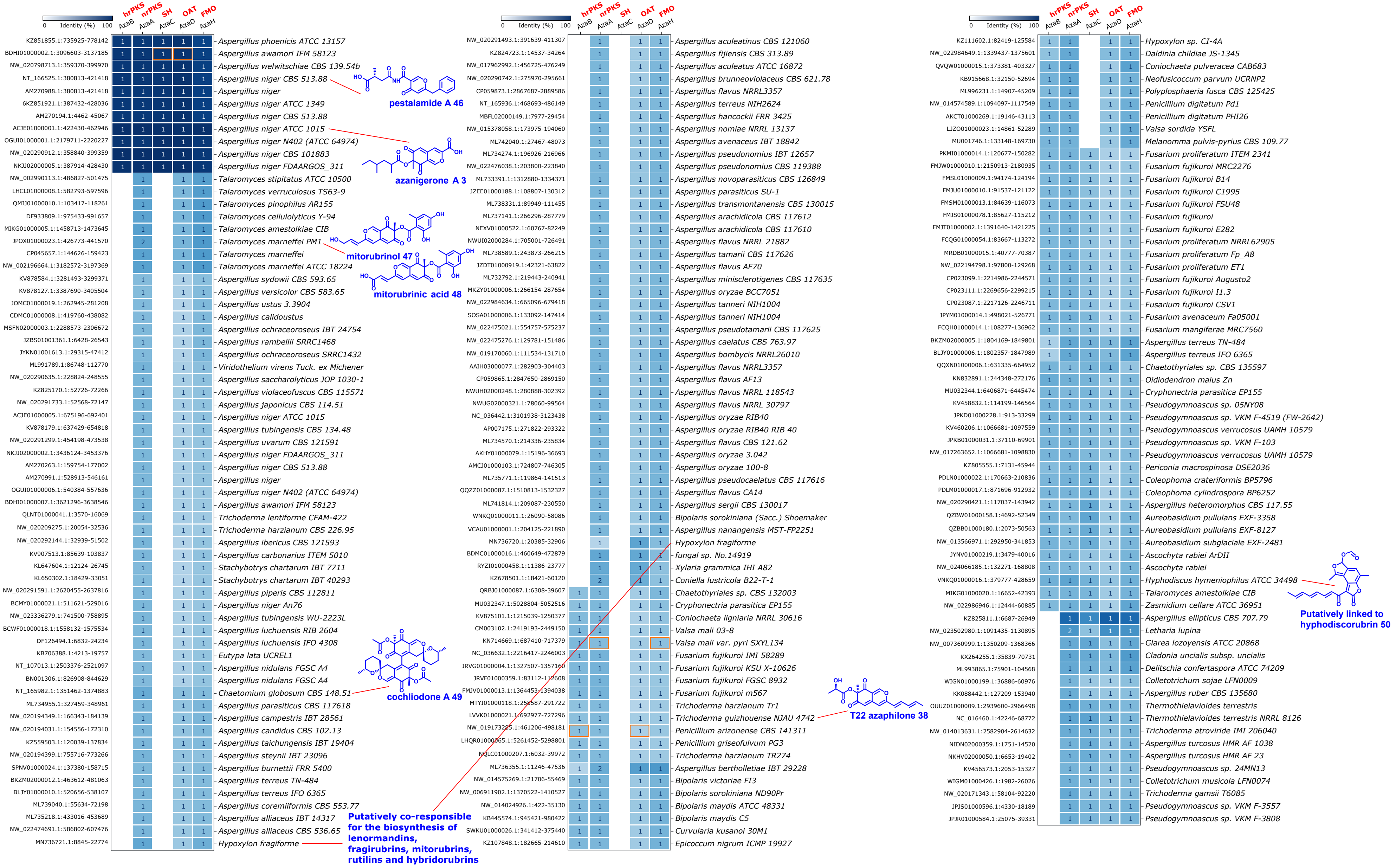

Figure S3: cblaster[4] search of annotated genomes on NCBI using 5 queries from the azaphilone BGC for azanigerone A biosynthesis (AzaB, AzaA, AzaC, AzaD and AzaH). The number within the box denotes the number of hits to a specific query sequence within the BGC. The orange cell border denotes multi hits of query sequences within the BGC. Search returned 24169 hits from 24091 unique sequences. cblaster detected 193 clusters across 19713 genomic scaffolds from 5059 organisms. Required sequences were AzaA, AzaD and AzaH. 8 BGCs definitively[5] [6] [7] [8] [9] and putatively[10] [11] linked to azaphilone based compounds are identified, leaving 185 orphan putative azaphilone BGCs.

References

[1] L. Liu, Z. Zhang, C.-L. Shao, J.-L. Wang, H. Bai, C.-Y. Wang, *Sci. Rep.* **2015**, *5*, 10463.
[2] S. Q. Le, O. Gascuel, *Mol. Biol. Evol.* **2008**, *25*, 1307-1320.
[3] S. Kumar, G. Stecher, M. Li, C. Knyaz, K. Tamura, *Mol. Biol. Evol.* **2018**, *35*, 1547-1549.
[4] C. L. M. Gilchrist, T. J. Booth, Y.-H. Chooi, **2020**, bioRxiv preprint DOI: 10.1101/2020.11.08.370601.
[5] A. O. Zabala, W. Xu, Y. H. Chooi, Y. Tang, *Chem. Biol.* **2012**, *19*, 1049-1059.
[6] B. Wang, X. Li, D. Yu, X. Chen, J. Tabudravu, H. Deng, L. Pan, *Microbiol. Res.* **2018**, *217*, 101-107.
[7] P. C. Y. Woo, C.-W. Lam, E. W. T. Tam, C. K. F. Leung, S. S. Y. Wong, S. K. P. Lau, K.-Y. Yuen, *PLoS Neglected Trop. Dis.* **2012**, *6*, e1871.
[8] T. Nakazawa, K. i. Ishiuchi, M. Sato, Y. Tsunematsu, S. Sugimoto, Y. Gotanda, H. Noguchi, K. Hotta, K. Watanabe, *J. Am. Chem. Soc.* **2013**, *135*, 13446-13455.
[9] G. Pang, T. T. Sun, Z. Z. Yu, T. Yuan, W. Liu, H. Zhu, Q. Gao, D. Q. Yang, C. P. Kubicek, J. Zhang, Q. R. Shen, *Environ. Microbiol.* **2020**, *22*, 4808-4824.
[10] K. Becker, S. Pfutze, E. Kuhnert, R. J. Cox, M. Stadler, F. Surup, *Chem. - Eur. J.* **2021**, *27*, 1438-1450.
[11] G. J. Kramer, S. Pimentel-Elardo, J. R. Nodwell, *ChemBioChem* **2020**, *21*, 2116-2120.
